# Distribution pattern of tumor associated macrophages predicts the prognosis of gastric cancer

**DOI:** 10.18632/oncotarget.21575

**Published:** 2017-10-06

**Authors:** Jiu-Yang Liu, Chun-wei Peng, Gui-Fang Yang, Wen-Qing Hu, Xiao-Jun Yang, Chao-Qun Huang, Bin Xiong, Yan Li

**Affiliations:** ^1^ Department of Gastrointestinal Surgery, Zhongnan Hospital of Wuhan University, Hubei Key Laboratory of Tumor Biological Behaviors & Hubei Cancer Clinical Study Center, Wuhan, China; ^2^ Department of Pathology, Zhongnan Hospital of Wuhan University, Wuhan, China; ^3^ Department of Surgery, The First Affiliated Hospital of Shanxi Medical University, Taiyuan, 030001, PR China; ^4^ Department of Peritoneal Cancer Surgery, Cancer Center of Beijing Shijitan Hospital Affiliated to the Capital Medical University, Beijing, China

**Keywords:** gastric cancer, tumor microenvironment, macrophages, prognosis

## Abstract

**Purpose:**

As mayor biomarkers in tumor microenvironment (TME), tumor associated macrophages (TAMs) of gastric cancer (GC) still needs further studies in terms of the number and distribution pattern.

**Methods:**

Herein, tissue microarrays (TMA) incorporating 494 GC surgical samples in duplicate were stained for TAMs infiltration analysis. TAMs number was counted according to the locations, including infiltrating macrophages in cancer nest (MC), in invasive front (MF) and in stroma (MS). Correlations between TAMs number, distribution pattern and clinic-pathological features and survival analyses were performed.

**Results:**

Infiltrating macrophages number in GC tissues was much higher than that in peritumoral tissues. TAMs number was not significantly correlated with the overall survival (OS). TAMs distribution pattern could be categorized into MC or MF/MS dominant pattern, and correlated with histological grade (*P* =0.001). The median OS of MF/MS dominant pattern (22.1, 95%CI: 23.5-28.9) was significantly shorter than that of MC dominant pattern (25.6, 95%CI: 28.5-35.6) (*P* =0.002). By receiver operating characteristic curve (ROC) analysis, the predictive value of TAMs distribution pattern was superior to histological grade and pM stage, but inferior to pN and TNM stage.

**Conclusions:**

TAMs distribution pattern could be an independent prognostic factor for the OS of GC patients, and patients with MF/MS dominant pattern had worse outcomes.

## INTRODUCTION

Tumor microenvironment (TME) plays an important role in cancer progression and metastasis [1, 2]. Within TME, distinct immune cells are recruited by cancer-derived signals and mutually interact with cancer cells [3]. Tumor associated macrophages (TAMs), the most abundant immune-related stromal cells [4], act as key orchestrators in TME, by directly attacking cancer cells, or promoting cancer progression by suppressing antitumor immunity, or inducing angiogenesis [5].

Significant advances have been made in TAMs studies regarding their impacts on clinical outcomes. The clinical significance of TAMs can be influenced by the number, phenotypes and distributions at each pathological stage [6]. Studies in gastric cancer (GC) have shown that higher number of TAMs is associated with worse prognosis [7, 8]. However, other reports have found that a higher level of TAMs infiltration results in a better outcome [9]. Therefore, it becomes controversial that TAMs emerge as significant but opposite predictors of survival for GC [10, 11]. These conflicting results could be due to the fact that most studies pay attention to the ratios of TAMs with different phenotypic features [12, 13], or ignorance of TAMs distributions by simply focusing on the number.

TAMs at different locations within the tumor may have different impacts on GC progression [14]. TAMs infiltration into tumor stroma has significant clinical relevance in GC, indicating the importance of not only studying the number but also studying the locations [15]. TAMs infiltration at invasive front could influence cancer metastasis through epithelial-mesenchymal transition (EMT) mechanism [16]. In turn, the degree of cell-to-cell contact may also influence the balance between protumoral and antitumoral properties of macrophages [17]. Some studies have also suggested that GC TAMs in different locations play different roles in relation to angiogenesis, stromal reaction, and prognosis [18]. Taken together, these results indicate that TAMs number and distributions are crucial factors to impact the coevolution between cancer cells and TAMs.

Therefore, this study evaluated both the number and locations of TAMs, especially the distribution pattern. TAMs number was counted at three locations, including cancer nest (MC), invasive front (MF), and stroma (MS). By comparison of TAMs number, TAMs distribution pattern could be defined as MC and MF/MS dominant pattern. Correlations of TAMs number and distribution pattern with GC clinical outcomes were both evaluated.

## RESULTS

### Infiltrating macrophages in peritumoral and GC tissues

**Figure 1:**
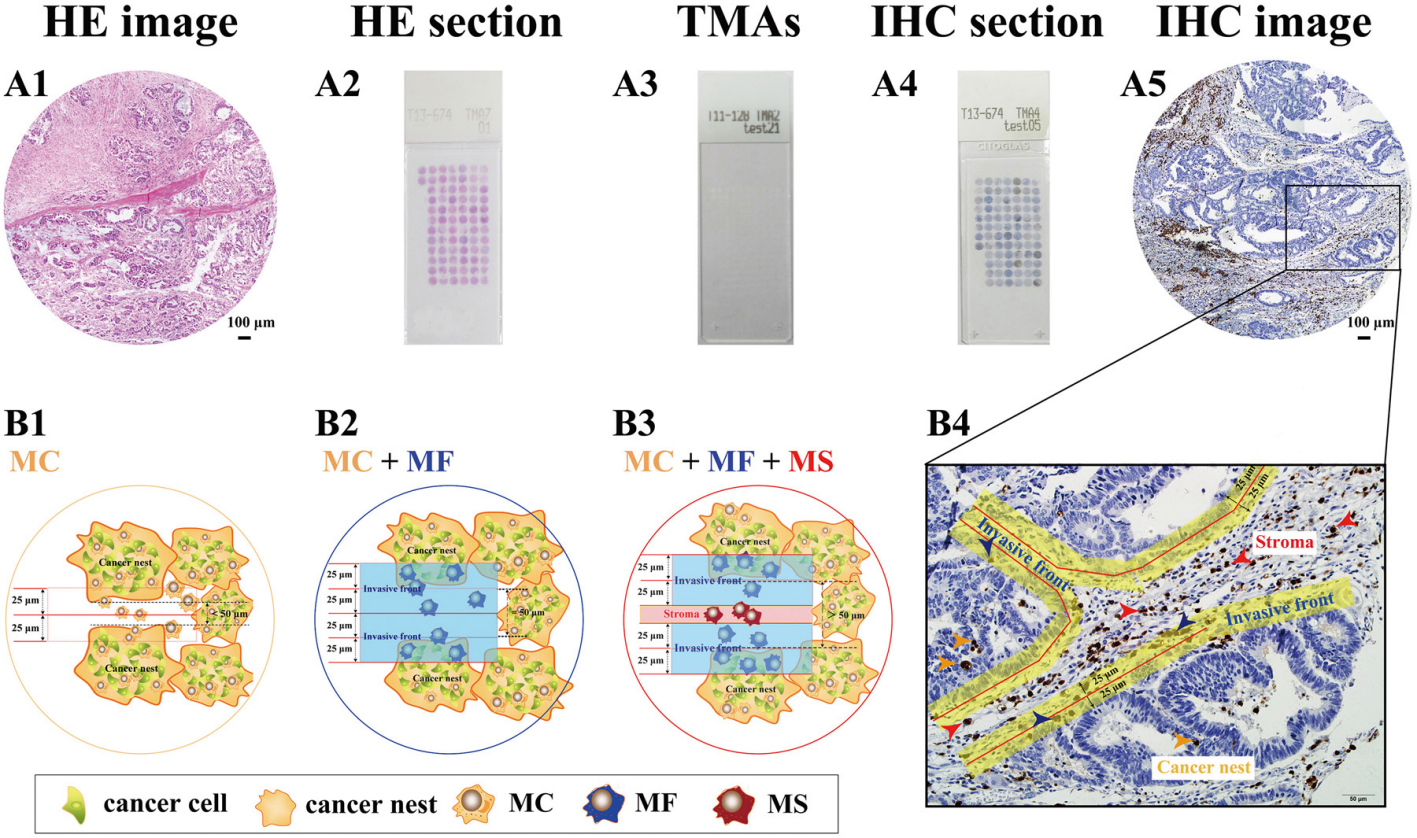
Major technical procedures and definitions of TAMs distribution patterns **(A)** Representative GC tissues were obtained for the establishment of tissue microarrays (TMAs) (A1-A3), and the immunohistochemistry experiments were performed well on TMAs (A4-A5). **(B)** The number and distribution of TAMs were analyzed and three TAMs locations including MC, MF, MS were found out. In virtual, one field could be divided into three parts, including cancer nest, cancer invasive front, and cancer stroma. The number of TAMs was evaluated for all locations. (B1) When the distance between two cancer nests were less than 50μm, such two cancer nests were considered just one big cancer nest. The TAMs in those areas were named as MC, MC=Total TAMs. (B2) The TAMs in stroma and nest which stay from the junction of stroma and nest less or equal to 25μm were named as MF. When the distance between two cancer nests were just equal to 50μm, there were only MC and MF, MC+MF =Total TAMs. (B3) When the distance between two cancer nests were more than 50μm, such two cancer nests were considered as two distinct cancer nests. The TAMs in stromal which stay from the neighboring cancer nest more than 25μm were named as MS. MC+MF+MS =Total TAMs. (B4) In this study, there were 296 patients could evaluate MC, MF and MS simultaneously.

**Figure 2:**
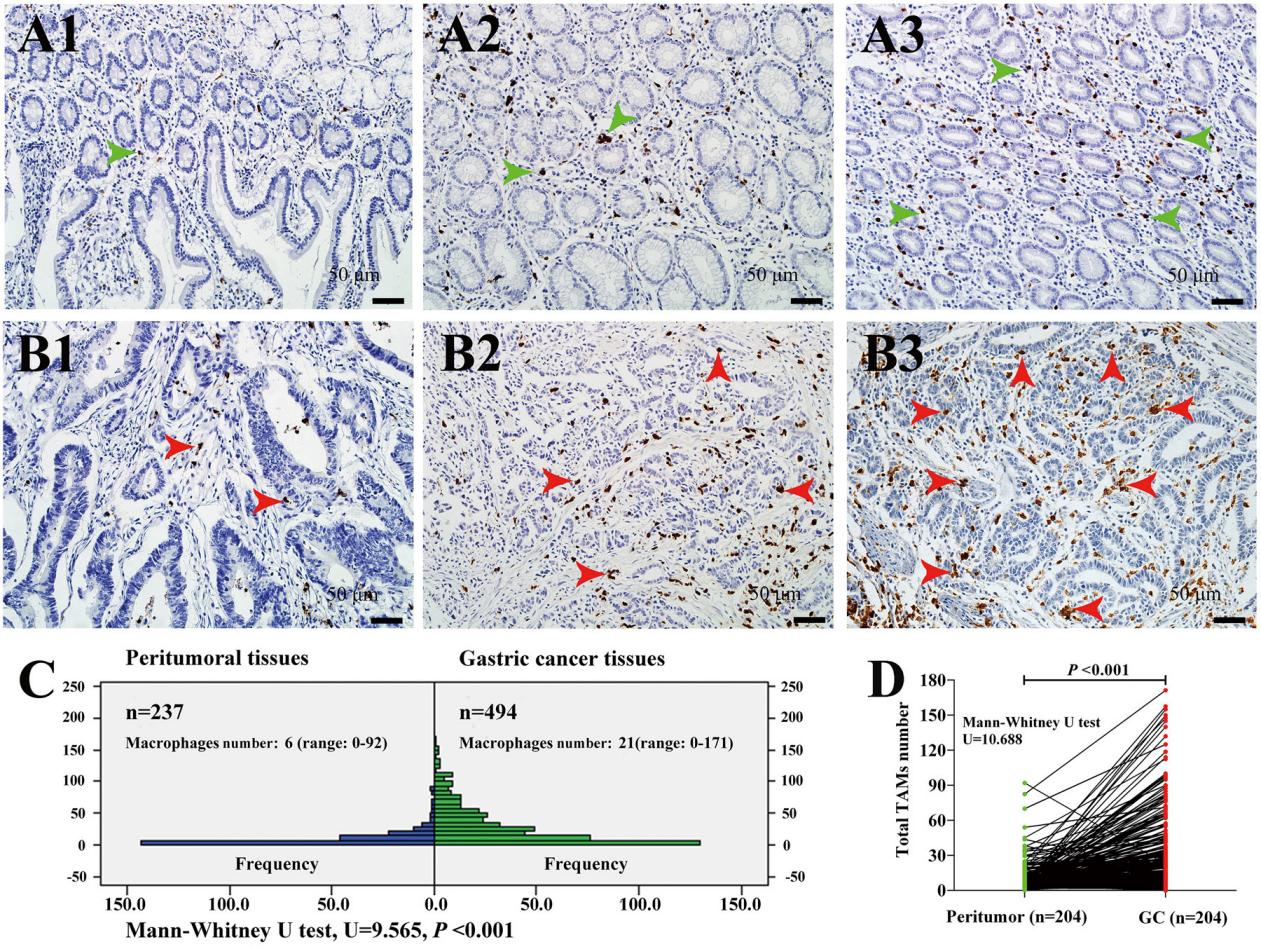
Infiltrating macrophages in peritumoral and GC tissues **(A)** Infiltrating macrophages in peritumoral tissues gradually increased in A1, A2 and A3. Green arrowheads indicated macrophages. **(B)** Infiltrating macrophages in GC tissues. Macrophages were thinly scattered in B1, accumulated in B2 and in large numbers in B3. Red arrowheads indicated tumor associated macrophages (TAMs). **(C)** The frequency distribution of infiltrating macrophages number in 237 peritumoral tissues and 494 GC tissues. **(D)** The self-comparison of 204 GC cases with relative peritumoral tissues. Magnification: 200×, Scale bar =50μm.

Results were obtained according to the flowchart in Figure 1. Infiltrating macrophages could be seen in both peritumoral (Figure 2A) and GC tissues (Figure 2B). The median number of infiltrating macrophages in peritumoral tissues (n=237) vs. GC tissues (n=494) was 5.5 (range: 0-92.0) vs. 21.0 (range: 0-171.3) per high power field (hpf) (*P* <0.001) (Figure 2C).

Among 204 GC cases in which TAMs presented both in cancer and peritumoral tissues, there was a trend towards significant higher number of TAMs in tumor tissues than peritumoral tissues (*P* <0.001) (Figure 2D).

### Correlations between total TAMs number and clinic-pathological features and OS

The relationship between total TAMs number and major clinic-pathological characteristics were studied in 494 GC patients (Table 1). Total TAMs number was significantly correlated with pathological types (*P* =0.003), serosa invasion (*P* =0.007) and TNM stage (*P* =0.009), but not significantly correlated with age, gender, tumor location, histological grade, lymph node metastasis, distant metastasis (*P* >0.05 for all).

**Table 1:** Major clinic-pathological characteristics and total TAMs number of GC patients

Variables	No. (%)	Total TAMs number		
		Median	Range	*P* ^a^
Age (Means ± SD, yrs)	59.0±11.9			
≤ 59	249 (50.4)	22	0-158	0.896
> 59	245 (49.6)	20	0-171	
Gender				
Male	349 (70.6)	19	0-155	0.073
Female	145 (29.4)	24	0-171	
Tumor location ^b^				
Distal stomach	227 (46.0)	21	0-171	0.965
Non-distal stomach	267 (54.0)	20	0-158	
Histological grade				
1/2	164 (33.2)	22	0-145	0.737
3/4	330 (66.8)	20	0-171	
Pathological types				
Well/Moderately differentiated adenocarcinoma	131 (26.5)	23	0-145	**0.003**
Low/Undifferentiated adenocarcinoma	284 (57.5)	23	0-171	
Mucinous adenocarcinoma/signet-ring cell carcinoma	66 (13.4)	13	0-105	
Others	13 (2.6)	9	0-78	
Serosa invasion				
No (T0, T1, T2)	104 (21.1)	27	0-158	**0.007**
Yes (T3, T4)	390 (78.9)	18	0-171	
Lymph node metastasis				
No (N0)	166 (33.6)	23	0-158	0.472
Yes (N1, N2, N3)	328 (66.4)	19	0-171	
Distant Metastasis				
M0	473 (95.7)	21	0-171	0.734
M1	21 (4.3)	31	0-126	
TNM stages				
Early (Stages I, II)	177 (35.8)	26	0-155	**0.009**
Advanced (Stages III, IV)	317 (64.2)	16	0-171	
Survive				
Yes	295 (59.7)	23	0-158	**<0.001**
No	199 (40.3)	18	0-171	

^a^ *P*-value in bold indicates that the difference was significant

^b^ Tumor location was classified according to the Japanese classification of gastric carcinoma (3rd English edition)

**Figure 3:**
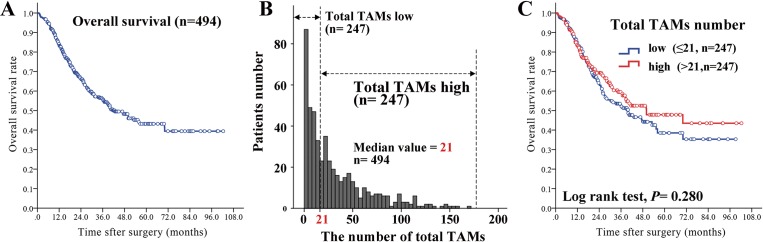
The prognostic value of total TAMs number **(A)** The median OS of 494 GC cases was 21.0 (range: 0.77-102.33) months. **(B)** Using the median value of total TAMs number as the threshold, there were 247 (50.0%) and 247 (50.0%) patients documented as total TAMs low and high number subgroup. **(C)** There was no significant difference regarding GC OS between total TAMs low and high number subgroups.

The median OS of 494 GC cases was 21.0 (95%CI: 25.2-28.7) months (Figure 3A). There were 199 (40.3%) dead, and the median OS was 15.0 (95CI: 16.6-20.3) months. Total TAMs number in survival group (median: 22.8, range: 0-157.5) was significantly higher than that in dead group (median: 17.5, range: 0-171.3) (*P* <0.001). According to the median value of total TAMs number, 494 patients could be classified into total TAMs -low (n=247) and -high subgroups (n=247). The difference in OS between two subgroups was not statistically significant (*P* =0.280) (Figure 3B and 3C).

### TAMs locations and distribution pattern

According to the criteria on TAMs locations, 494 patients could be evaluated for MC, 319 patients could be evaluated for both MC and MF, and 296 patients could be evaluated for all MC, MF and MS. Major clinic-pathological and survival information among the three databases were comparable (Supplementary Table 1). Detailed analyses were performed on 296 patients to study the impact of MC number, MF/MS number, and TAMs distribution pattern on GC prognosis.

**Figure 4:**
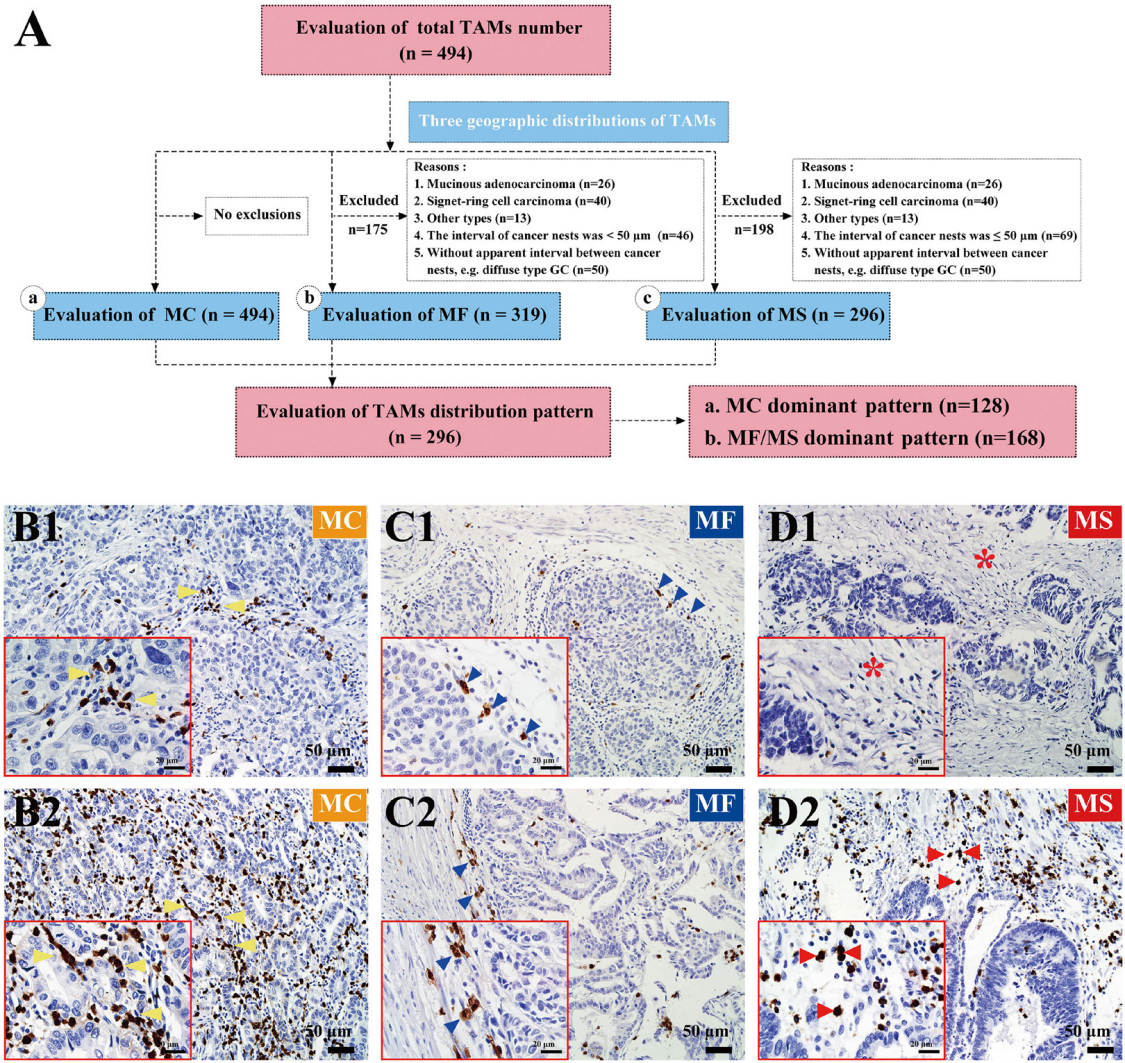
TAMs locations and distribution patterns **(A)** The flowchart and detailed exclusion criteria. **(B)** MC were diffusely distributed within cancer nest. Tumor epithelium-infiltrating macrophages were representative features to present spatial relationships between TAMs and cancer cells. Yellow arrowheads in B1 and B2 indicated MC with low and high number, respectively. **(C)** Blue arrowheads in C1 and C2 indicated MF with low and high number, respectively. **(D)** MS were mostly seen in well and moderately differentiated adenocarcinoma. Red stars in D1 showed stromal regions with no TAMs infiltration. Red arrowheads in D2 indicated MS with high number. Magnification: ×200, Scale bar =50μm. Photos in red frames, Magnification: ×400, Scale bar =20μm.

The flowchart and detailed exclusion criteria was shown in Figure 4A. Representative photos of MC, MF, and MS were shown in Figure 4B-4D.

### Correlations between MC, MF/MS number and clinic-pathological features and OS

Out of 296 GC patients, correlations between total TAMs number, MC number, MF/MS number, and major clinic-pathological characteristics were studied (Table 2). The median value of total TAMs number was 25 (range: 0-171). Total TAMs number was significantly correlated with serosa invasion (*P* =0.019), but not significantly correlated with age, gender, tumor location, histological grade, lymph node metastasis, distant metastasis, and TNM stage (*P* >0.05 for all). MC number was significantly correlated with tumor location (*P* =0.018), and histological grade (*P* =0.007), but not significantly correlated with age, gender, serosa invasion, lymph node metastasis, distant metastasis (*P* >0.05 for all). MF/MS number (median value: 13, range: 0-166) was higher than MC number (median value: 6, range: 0-126), the difference was statistically significant (*P* <0.001). MF/MS number was significantly correlated with histological grade (*P* =0.045), and lymph node metastasis (*P* =0.037), but not significantly correlated with age, gender, tumor location, serosa invasion, distant metastasis, and TNM stage (*P* >0.05 for all).

**Table 2:** Major clinic-pathological characteristics and TAMs number, distribution pattern in 296 GC patients

Variables	No. (%)	Total TAMs number			MC number			MF/MS number			TAMs distribution pattern (n, %)		
		Median	Range	*P* ^a^	Median	Range	*P*^a^	Median	Range	*P*^a^	MC	MF/MS	*P*^a^
Age (Means ± SD, yrs)	59.9±11.7												
≤ 59	145 (49.0)	28	0-171	0.352	8	0-90	0.201	12	0-160	0.563	63 (21.3)	82 (27.7)	1.000
> 59	151 (51.0)	22	0-171		5	0-126		14	0-160		65 (22.0)	86(29.0)	
Gender													
Male	218 (73.6)	24	0-171	0.513	6	0-95	0.943	13	0-114	0.359	92 (31.1)	126 (42.5)	0.595
Female	78 (26.4)	26	1-171		6	0-126		18	0-166		36 (12.2)	42 (14.2)	
Tumor location ^b^													
Upper third	95 (32.1)	23	0-171	0.145	6	0-95	**0.018**	14	0-110	0.545	36 (12.2)	59 (19.9)	0.473
Middle third	75 (25.3)	30	1-166		11	0-90		16	0-166		37 (12.5)	38 (12.8)	
Lower third	115 (38.9)	23	0-171		6	0-126		13	0-160		51 (17.2)	64 (21.7)	
Total stomach	11 (3.7)	15	3-101		4	0-39		7	0-80		4 (1.3)	7 (2.4)	
Histological grade													
1/2	124 (41.9)	25	0-136	0.906	4	0-85	**0.007**	19	0-120	**0.045**	40 (13.5)	84 (28.4)	**0.001**
3/4	172 (58.1)	24	0-171		9	0-126		11	0-166		88 (29.7)	84 (28.4)	
Serosa invasion													
No (T0, T1, T2)	68 (23.0)	31	1-171	**0.019**	8	0-115	0.582	20	0-160	0.053	27 (9.1)	41 (13.9)	0.577
Yes (T3, T4)	228 (77.0)	21	0-171		6	0-126		12	0-166		101 (34.2)	127 (42.8)	
Lymph node metastasis													
No (N0)	100 (33.8)	29	0-171	0.111	7	0-78	0.806	16	0-110	**0.037**	38 (12.9)	62 (20.9)	0.216
Yes (N1, N2, N3)	196 (66.2)	22	0-171		6	0-126		11	0-166		90 (30.4)	106 (35.8)	
Distant Metastasis													
M0	283 (95.6)	25	0-171	0.783	7	0-126	0.085	13	0-160	0.783	123 (41.6)	160 (54.0)	0.782
M1	13 (4.4)	26	1-166		3	0-84		14	0-166		5 (1.7)	8 (2.7)	
TNM stage													
Stages I	45 (15.2)	30	2-104	0.338	8	0-50	0.296	18	0-100	0.187	16 (5.4)	29 (9.8)	0.704
Stages II	67 (22.6)	26	0-171		6	0-90		15	0-160		29 (9.8)	38 (12.8)	
Stages III	172 (58.1)	20	0-171		7	0-126		11	0-120		78 (26.3)	94 (31.8)	
Stages IV	12 (4.1)	25	1-166		3	0-84		11	0-166		5 (1.7)	7 (2.4)	
Survive													
Yes	182 (61.5)	25	0-171	0.812	8	0-115	0.284	13	0-120	0.551	90 (30.4)	92 (31.1)	**0.008**
No	114 (38.5)	22	0-171		5	0-126		14	0-166		38 (12.8)	76 (25.7)	

^a^ *P*-value in bold indicates that the difference was statistically significant

^b^ Tumor location was classified according to the Japanese classification of gastric carcinoma (3rd English edition)

Correlations between total TAMs number, MC number, MF/MS number and GC OS were also investigated. According to the median value of macrophages number, 296 patients were classified as total TAMs -low (n =150) and -high (n =146), as MC -low (n =149) and -high (n =147), as MF/MS -low (n =150) and -high (n =146). No significant survival differences were observed regarding total TAMs number (*P* =0.583), MC number (*P* =0.544) and MF/MS number (*P* =0.104).

### Correlations between TAMs distribution pattern and clinic-pathological features and OS

Among 296 GC patients, there were 128 (43.3%) cases in which MC number (median: 12, range: 0-126) was significantly higher than MF/MS number (median: 4, range: 0-75) (*P* <0.001). These cases were defined as MC dominant pattern. Similarly, there were 168 (56.7%) cases in which MF/MS number (median: 23, range: 1-166) was significantly higher than MC number (median: 4, range: 0-73) (*P* <0.001). These patients were defined as MF/MS dominant pattern.

**Figure 5:**
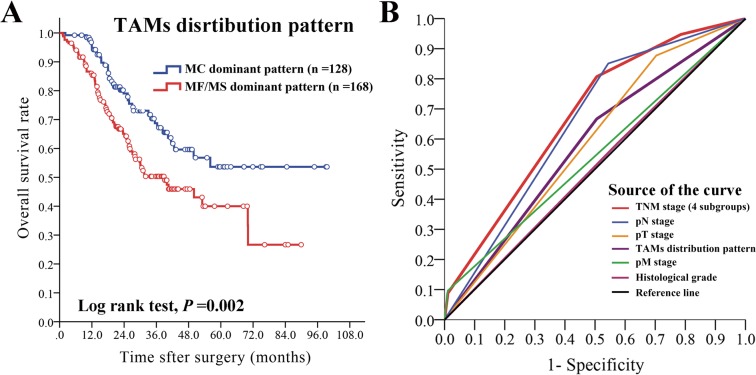
The prognostic value of TAMs distribution pattern and ROC analysis **(A)** The prognostic value of TAMs distribution pattern. The median OS of patients with MF/MS dominant pattern was shorter than that of patients with MC dominant pattern, the differences was statistically significant (*P*=0.002). **(B)** ROC analysis indicated that TAMs distribution pattern (AUC: 0.581 [95%CI: 0.514-0.647], *P* =0.020) was superior to histological grade and pM stage, but inferior to pN stage and TNM stage.

Compared with MC dominant pattern, MF/MS dominant pattern was significantly correlated with histological grade (*P* =0.001), pathological types (*P*=0.004), but not significantly correlated with age, gender, tumor location, serosa invasion, lymph node metastasis, distant metastasis and TNM stage (*P* >0.05 for all) (Table 2). The median OS of MF/MS dominant pattern (22.1, 95%CI: 23.5-28.9) was significantly shorter than that of MC dominant pattern (25.6, 95%CI: 28.5-35.6) (*P* =0.002, Figure 5A).

### Uni- and multivariate analysis and ROC analysis of TAMs distribution pattern

The univariate analysis of clinic-pathological factors and TAMs distribution pattern regarding OS was also conducted. In univariate analysis, traditional clinic-pathological features (such as histological grade, pT stage, pN stage and TNM stage), and MF/MS distribution pattern (*P* =0.002) were associated with OS (Table 3).

**Table 3:** Analyses of clinic-pathological factors and TAMs distribution pattern regarding OS in 296 GC patients

Variables	No. (%)	No. of death (%)	3-year survival rate	5-year survival rate	Log-rank test χ^2^ value	P^*^
Histological grade						
1/2	124 (41.9)	47 (37.9)	57.36%	45.52%	0.104	0.747
3/4	172 (58.1)	67 (39.0)	57.80%	44.88%		
T stage						
T1-2	68 (23.0)	14 (20.6)	71.06%	66.62%	8.727	**0.003**
T3-4	228 (77.0)	100 (43.9)	54.02%	39.86%		
Lymph nodes metastasis						
No	100 (33.8)	17 (17.0)	76.00%	73.14%	25.330	**<0.001**
Yes	196 (66.2)	97 (49.5)	48.98%	33.00%		
Distant Metastasis						
M0	283 (95.6)	103 (36.4)	60.01%	46.90%	21.956	**<0.001**
M1	13 (4.4)	11 (84.6)	13.19%	13.19%		
TNM stage						
Stages I	45 (15.2)	6 (13.3)	78.94%	78.94%	37.833	**<0.001**
Stages II	67 (22.6)	16 (23.9)	71.70%	63.00%		
Stages III	172 (58.1)	82 (47.7)	50.90%	34.90%		
Stages IV	12 (4.1)	10 (83.3)	14.29%	14.29%		
TAMs distribution pattern	296 (100)	114 (38.5)	57.60%	45.06%	9.722	**0.002**
MC dominant pattern	128 (43.2)	38 (29.7)	68.21%	53.24%		
MF/MS dominant pattern	168 (56.8)	76 (45.2)	49.85%	38.78%		

^*^: *P*-value in bold indicates that the difference was significant.

These factors were also integrated into multivariate Cox proportional hazards analysis. In multivariate analysis, significant factors correlating with OS were pN stage (*P* =0.008), pM stage (*P* =0.020), TNM stage (*P* <0.001), and TAMs distribution pattern (hazard ratio, HR =2.177 [95%CI: 1.449-3.270], *P* <0.001) (Table 4).

**Table 4:** Multivariate analysis and ROC analysis of factors associated with OS

Factors	OS			ROC analysis		
	HR	95%CI	P^*^	Area under curve (AUC)	95% CI	P^*^
Histological grade				0.505	0.438-0.573	0.876
1/2	1.000					
3/4	0.971	0.661-1.436	0.894			
T stage				0.587	0.522-0.652	**0.012**
T0, 1, 2	1.000		0.071			
T3, 4	3.352	0.903-12.437				
N stage				0.653	0.591-0.716	**<0.001**
N0	1.000		**0.008**			
N1, 2, 3	6.073	1.590-23.192				
M stage				0.543	0.474-0.611	0.216
M0	1.000		**0.020**			
M1	17.034	1.568-185.083				
TNM stage				0.676	0.615-0.737	**<0.001**
Stage I	1.000					
Stage II	1.677	0.654-4.297	0.281			
Stage III	4.007	1.746-9.199	**0.001**			
Stage IV	10.880	3.929-30.130	**<0.001**			
TAMs distribution pattern				0.581	0.514-0.647	**0.020**
MC dominant	1.000					
MF/MS dominant	2.177	1.449-3.270	**<0.001**			

^*^: *P*-value in bold indicates that the difference was significant.

ROC analysis was conducted to further evaluate the prognostic performance of histological grade, pT stage, pN stage, pM stage, TNM stage, and MF/MS distribution pattern. As shown in Figure 5B, the area under the curve (AUC) was the largest for TNM stage [AUC = 0.676, 95%CI: 0.615-0.737] (*P* <0.001), followed by pN stage [AUC = 0.653, 95%CI: 0.591-0.716] (*P* <0.001), pT stage [AUC = 0.587, 95%CI: 0.522-0.652] (*P* =0.012), TAMs distribution pattern [AUC = 0.581, 95%CI: 0.514-0.647] (*P* =0.020), pM stage [AUC = 0.543, 95%CI: 0.474-0.611] (*P* =0.216), and histological grade [AUC =0.505, 95%CI: 0.438-0.573] (*P* =0.876).

## DISCUSSION

Inflammation is one of the significant hallmarks of cancer, and influences the coevolution between cancer cells and TME [19]. Within the TME, TAMs recruited by tumor-derived signals might be the most important inflammation cells [20, 21]. Evaluating both the number and distributions could promote a comprehensive understanding of TAMs significance during GC progression.

In this study, TAMs number in GC tissues was much higher than that in peritumoral tissues. This result, in accordance with some previous studies, proved that TAMs were recruited and aggregated within tumor tissues, then mutually interacted with tumor cells [22]. In fact, TAMs also correlated with tumor progression. TAMs number had been identified as an independent prognostic factor in several cancer types, such as breast cancer and colorectal cancer [23, 24]. Herein, total TAMs number was higher in GC patients with no serosa invasion, early TNM stages, and in survival patients, which indicated TAMs may be protective factors of GC. To investigate the exact prognostic significance of TAMs, detailed analyses were focused on total TAMs number and OS, and no correlations were found. Such contradictory results have also been reported previously [25, 26]. Some studies considered TAMs as protumoral through induction of angiogenesis and suppression of antitumor immunity [27], while other reports found that a dense TAMs infiltration positively influenced prognosis in GC [9]. To explore such contradiction, we studied TAMs from aspects of both the number and histological distributions.

Based on the spatial position relationships between TAMs and tumor cells, MC and MF/MS number were counted and analyzed. The distribution characteristic of TAMs had also been mentioned previously [28, 29]. Su et al. elucidated mutual interactions between cancer cells and TAMs at tumor invasive front [28]. Additionally, the close vicinity of TAMs and tumor neo-vessels was observed in tumor stroma, and constituted tumor invasion unit [29]. On the basis of these morphological studies, quantitative counting analyses of TAMs were further conducted among different sites in GC. It turned out that MF/MS number was much higher than MC number, showing that TAMs tended to locate at invasive front and in stroma. However, no significant relationships could be reported between total TAMs number, MC number or MF/MS number and GC prognosis in this study. Therefore, simply analyzing TAMs number with ignorance of distribution locations was not sufficient [30]. Taken together, TAMs number might not be a significant prognostic marker related to GC OS.

Herein further analysis was developed using TAMs distribution pattern through a definition by systematical integration of TAMs number and histological distributions, which is crucial to reflect the coevolution between tumor cells and TME [31]. In this study, GC patients with MF/MS dominant pattern had worse clinical outcomes than MC dominant pattern, indicating MF/MS as main contributors to promote GC progression. Moreover, the predictive value of TAMs distribution pattern was verified by ROC analysis. Theoretically, the heterogeneity and plasticity were hallmarks of macrophages [32]. TAMs could undergo phenotypic switch from the classically activated macrophages (also known as M1 macrophages) to the alternatively activated macrophages (M2 macrophages). M1 macrophages were described as pro-inflammatory to display tumor-resistant effects, while M2 macrophages often associated with tumor-promoting properties. Consequently, TAMs gained potentials to promote cancer cell motility in invasion areas, to promote metastasis in stromal and perivascular areas, and to stimulate angiogenesis in avascular and peri-necrotic hypoxic areas [33]. Therefore, TAMs among different sites in GC tissues might represent distinct significances and prognostic values [34]. Owing to vigorous cell-to-cell contacts among TAMs, cancer cells and other activated stromal cells, TAMs along tumor invasive front are of great significance. At tumor invasive front, TAMs might correlate with various signaling pathways [35], undergo phenotypically switch from M1 to M2 [36], and finally promote the acquisition of specific pathological features of cancers such as immunosuppression, neovascularization and modification of extracellular matrix [37]. Pinto et al. have shown that TAMs with M2 phenotype were recruited by multiple chemoattractant and accumulated at the interface between cancer nest and stroma [38], thereby raising a hypothesis that the main constituent of MF/MS was M2 macrophages, which had been verified in some studies and correlated with poor prognosis [39].

With these in consideration, MF/MS could be a predominant and decisive factor, while MC might be a less important factor on GC prognosis. TAMs distribution pattern integrated both MC and MF/MS into consideration, and could be an independent prognostic factor of GC. The advantage of TAMs distribution pattern was due to the significance of researching TME and complex quantitative analyses [40]. However, limitations also existed by optimizing single biomarker, thus demonstrating TAMs distribution pattern inferior to TNM stage. To make sense of the overall landscape of TAMs, combined analyses with other biomarkers may provide new insights for deep understandings [41].

In summary, TAMs number merely correlates with several unfavorable clinic-pathological features, but has no significant prognostic value. Both the number and distributions should be taken into consideration for TAMs evaluations. Promisingly, TAMs distribution pattern could be a new factor related to GC OS.

## MATERIALS AND METHODS

### Study population and database

The records of patients who underwent surgical resection of GC from December 2002 to February 2011 were reviewed. Major demographic and clinic-pathological characteristics were retrieved. The tumor type, histologic grade, depth of invasion, number of lymph nodes retrieved, and number of lymph nodes with metastases were re-confirmed histologically. Inclusion and exclusion criteria were defined as follows. Patients were included when histology confirmed adenocarcinoma of the stomach and the survival data were available. Patients were excluded when distant metastasis has been diagnosed before surgery, histology identified a pathological type other than adenocarcinoma, R1 resection, no lymph node was retrieved or histopathological and survival data were incomplete. No patients receive neoadjuvant chemotherapy. In this study, 329 (66.6%) patients received adjuvant chemotherapy. In stage III patients (n = 316), 217 (68.7%) patients received adjuvant chemotherapy, and only 69 patients received more than six cycles of adjuvant chemotherapy. TNM stage was determined according to the 7th edition UICC/AJCC TNM staging system. Overall survival (OS), defined as the duration from operation to GC-related death or last follow-up, was used for prognosis evaluation. The primary endpoint of this study was OS, and patients alive at the last follow-up were recorded as censored events.

### Ethics statement

Written informed consent was obtained from the patients with the study protocol approved by the ethics committee of Zhongnan Hospital of Wuhan University. The study was undertaken in accordance with the ethical standards of the World Medical Association Declaration of Helsinki.

### Tissue microarrays (TMAs) and immunohistochemistry (IHC)

TMAs have been constructed for this study. Briefly, two cores were taken from each representative tumor tissue and peritumoral tissue (at least 50mm away from the tumor border). Then, sixteen TMAs sections with 494 tumor tissues (988 cores, 2mm each core) and 237 peritumoral tissues (474 cores, 2mm each core) were constructed (in collaboration with Shanghai Biochip Company Ltd., Shanghai, China).

Routine IHC method was performed for the staining of TAMs. The primary antibody was mouse anti-human monoclonal antibody against macrophages (ab22506 [MAC387], Abcam, UK, dilution 1/100), with corresponding horseradish peroxidase (HRP) conjugated secondary antibody (ab97265, Abcam, UK, dilution 1/300). The reaction products were visualized with diaminobenzidine (DAB, DAKO, Denmark). Then the slides were evaluated by two senior pathologists, who were blinded to the patients’ clinical features and outcomes. A consensus was achieved using a multi-headed microscope in case of discrepancy. In brief, one TMA core with at least 4 standard-compliant vision fields (magnification, ×200) per patient was considered to be adequate, with no focus on hotspots.

### Image acquisition and the classification criteria of TAMs locations and distribution patterns

The digital images of infiltrating macrophages staining were captured under Olympus BX51 fluorescence microscope equipped with Olympus DP72 camera (Olympus Optical Co., Ltd., Tokyo, Japan) at ×200 magnification. Identical settings were used for every photograph, so as to minimize the selection bias.

To assess the role of TAMs in GC progression, the number and the distribution of TAMs were evaluated. First, the positive cells were counted in at least four high power fields (hpfs, ×200 magnification) each core, and the average number of two cores (eight hpfs) from the same patient were recorded as the number of TAMs. Second, TAMs geographic distributions were assessed, aiming to uncover the exact role of TAMs in different areas. Theoretically, each field could be divided into three different regions including cancer nest, cancer invasive front and cancer stromal. Correspondingly, the TAMs in GCs could be classified into three distinct patterns including infiltrating macrophages in cancer nest (MC), infiltrating macrophages in invasive front (MF) and infiltrating macrophages in stromal (MS). Such classification represented the spatial position relationship between macrophages and cancer cells.

Then, the number of MC, MF and MS were recorded, respectively. Briefly, MC+MF+MS=Total TAMs. Notable, MF and MS may be zero. Actually, not each patient has three locations simultaneously, but may have one superiority location. Thus, some patients may have MC only, some patients may have MC and MF, and some patients may have MC, MF and MS simultaneously. In this study, there were 494 patients could be evaluated for MC, 319 patients could be evaluated for both MC and MF, and only 296 patients could be evaluated for all MC, MF and MS. Finally, total of 296 patients containing three locations of TAMs were classified into two distribution patterns, including MC dominant pattern and MF/MS dominant pattern. Investigators were blind to the clinic-pathological data and clinical outcomes of GC patients. Cut points of TAMs number for subgroup analysis were explored by the median value.

### Statistical analysis

Statistical analyses were performed with IBM SPSS 19.0 software (SPSS Institute, Chicago, IL). The correlations between the number of infiltrating TAMs and clinic-pathological parameters were calculated with the chi-square test. Survival probabilities between subgroups were analyzed with the Kaplan-Meier method, by use of the log-rank test for univariate analyses, and by use of Cox regression model for multivariate analyses. ROC analysis was used to determine the predictive value of the parameters. Two-sided *P* value of <0.05 was considered to be statistically significant.

## SUPPLEMENTARY MATERIALS TABLE



## Abbreviations

GC: gastric cancer
TME: tumor microenvironment
TAMs: tumor associated macrophages
IHC: immunohistochemistry
MC: macrophages in cancer nest
MF: macrophages in invasive front
MS: macrophages in stroma
OS: overall survival
HR: hazard ratio
CI: confidence interval
ROC: receiver operating characteristic curve
AUC: area under the curve.
