# Supplementary material for: Distribution pattern of tumor associated macrophages predicts the prognosis of gastric cancer
Source: Oncotarget. 2017 Oct 6;8(54):92757–69. doi: 10.18632/oncotarget.21575 (PMC5696219; doi:10.18632/oncotarget.21575)
Supplement: Supplementary file 1 [file oncotarget-08-92757-s001.pdf]

Distribution pattern of tumor associated macrophages predicts the prognosis of gastric cancer

SUPPLEMENTARY MATERIALS

Supplementary Table 1: Major clinic-pathological and survival information among three databases

Variables	Database 1 (n=494)	Database 2 (n=319)	Database 3 (n=296)
Age (Means ± SD, yrs)	59.0±11.9	59.9±10.9	59.6±10.8
≤ 59	249 (50.4)	156 (48.9)	145 (49.0)
> 59	245 (49.6)	163 (51.1)	151 (51.0)
Gender			
Male	349 (70.6)	236 (74.0)	218 (73.6)
Female	145 (29.4)	83 (26.0)	78 (26.4)
**Tumor location** [a]			
Distal stomach	227 (46.0)	134 (42.0)	126 (42.6)
Non-distal stomach	267 (54.0)	185 (58.0)	170 (57.4)
Histological grade			
1/2	164 (33.2)	132 (41.4)	124 (41.9)
3/4	330 (66.8)	187 (58.6)	172 (58.1)
Pathological types			
Well/Moderately differentiated adenocarcinoma	131 (26.5)	102 (32.0)	96 (32.4)
Low/Undifferentiated adenocarcinoma	284 (57.5)	184 (57.7)	169 (57.1)
Mucinous adenocarcinoma/signet-ring cell carcinoma	66 (13.4)	25 (7.8)	24 (8.1)
Others	13 (2.6)	8 (2.5)	7 (2.4)
Serosa invasion			
No (T0, T1, T2)	104 (21.1)	74 (23.2)	68 (23.0)
Yes (T3, T4)	390 (78.9)	245 (76.8)	228 (77.0)
Lymph node metastasis			
No (N0)	166 (33.6)	106 (33.2)	100 (33.8)
Yes (N1, N2, N3)	328 (66.4)	213 (66.8)	196 (66.2)
Distant Metastasis			
M0	473 (95.7)	306 (95.9)	283 (95.6)
M1	21 (4.3)	13 (4.1)	13 (4.4)
TNM stages			
Early (Stages I, II)	177 (35.8)	118 (37.0)	112 (37.8)
Advanced (Stages III, IV)	317 (64.2)	201 (63.0)	184 (62.2)
OS (Median, 95%CI)	21.0 (25.2-28.7)	23.6 (26.7-31.1)	23.6 (26.6-30.9)
Survive			
Yes	295 (59.7)	196 (61.4)	182 (61.5)
No	199 (40.3)	123 (38.6)	114 (38.5)

[a] Tumor location was classified according to the Japanese classification of gastric carcinoma (3rd English edition).
